# American Dental Association

**Published:** 1877-09

**Authors:** 


### AMERICAN DENTAL ASSOCIATION.

The annual meeting of this body was held in Chicago, August 7th to 10th. There was about one hundred members present, and quite a large number of visitors. There were papers presented and read on almost every topic in the programme, and some of them were very good, indeed, better than usual; the discussions, too, were for the most part interesting and profitable.

There was less time spent in matters of mere business detail than usual, and yet there was too much even on this occasion. There is sometimes too much enthusiasm expended on matters of very little importance.

A proposition was submitted for a change in the organization of the association, affecting more particularly the standing committees. This change contemplates chiefly the substitution of sections instead of standing committees; the sections to be composed of those who may volunteer to become members of these sections. Each division will have a special subject, which will be by it thoroughly considered and developed for presentation to the association, and it is presumed that subjects in a half digested form will not be presented.

Each section will select its chairman and such other officers as may be requisite for the proper performance of its work. It is intended that these sections shall be permanent in their organization, though changes will necessarily often occur in the membership of each. There will be an opportunity, and indeed it will be desirable, for every member of the association to have a place upon one or more of these sections.

This movement also contemplates a union of this body with the American Dental Convention, and the Southern Dental Association, which, we doubt not, will be a matter of gratification to every true friend and well-wisher of the profession in the country.

It is also proposed to place the representation upon rather a different basis than hitherto; viz: to receive delegates only from State Societies.

A committee of ten was appointed, to whom was assigned the duty of further considering and arranging for the completion of the union and organization.

If the changes, to which reference is made above, are carried out, it will doubtless produce quite a marked effect upon the profession. The work of the body in its sections will certainly be far better done than it has usually been by the standing committees, and the association will possess greater influence and power for good than heretofore. It will also serve as a stimulus for the organization and improvement of State Societies. We anticipate great things of the American Dental Association under such an organization. Dr. F. H. Rehwinkel was elected President. The next meeting will be held at Niagara Falls.

